# Interferon-Beta Therapy of Multiple Sclerosis Patients Improves the Responsiveness of T Cells for Immune Suppression by Regulatory T Cells

**DOI:** 10.3390/ijms160716330

**Published:** 2015-07-17

**Authors:** Bettina Trinschek, Felix Luessi, Catharina C. Gross, Heinz Wiendl, Helmut Jonuleit

**Affiliations:** 1 Department of Dermatology, University Medical Center of the Johannes Gutenberg-University, Langenbeckstr. 1, 55131 Mainz, Germany; E-Mail: bettina.trinschek@unimedizin-mainz.de; 2 Department of Neurology, University Medical Center of the Johannes Gutenberg-University, Langenbeckstr. 1, 55131 Mainz, Germany; E-Mail: luessi@uni-mainz.de; 3 Department of Neurology-Inflammatory Disorders of the Nervous System and Neurooncology, University of Muenster, Schlossplatz 2, 48149 Muenster, Germany; E-Mails: catharina.gross@ukmuenster.de (C.C.G.); heinz.wiendl@ukmuenster.de (H.W.)

**Keywords:** multiple sclerosis, therapy, diagnosis, immune regulation, T effector cells, Treg

## Abstract

Multiple sclerosis (MS) is an inflammatory autoimmune disease characterized by imbalanced immune regulatory networks, and MS patient-derived T effector cells are inefficiently suppressed through regulatory T cells (Treg), a phenomenon known as Treg resistance. In the current study we investigated T cell function in MS patients before and after interferon-beta therapy. We compared cytokine profile, responsiveness for Treg-mediated suppression *ex vivo* and evaluated reactivity of T cells *in vivo* using a humanized mouse model. We found that CD4^+^ and CD8^+^ T cells of therapy-naive MS patients were resistant to Treg-mediated suppression. Treg resistance is associated with an augmented IL-6 production, enhanced IL-6 receptor expression, and increased PKB/c-Akt phosphorylation. These parameters as well as responsiveness of T cells to Treg-mediated suppression were restored after interferon-beta therapy of MS patients. Following transfer into immunodeficient mice, MS T cells induced a lethal graft *versus* host disease (GvHD) and in contrast to T cells of healthy volunteers, this aggressive T cell response could not be controlled by Treg, but was abolished by anti-IL-6 receptor antibodies. However, magnitude and lethality of GvHD induced by MS T cells was significantly decreased after interferon-beta therapy and the reaction was prevented by Treg activation *in vivo*. Our data reveals that interferon-beta therapy improves the immunoregulation of autoaggressive T effector cells in MS patients by changing the IL-6 signal transduction pathway, thus restoring their sensitivity to Treg-mediated suppression.

## 1. Introduction

Multiple Sclerosis (MS) is an inflammatory autoimmune disease resulting from an imbalanced immune tolerance network. Crucial players of this network are autoreactive T effector cells usually kept in balance by regulatory T cells (Treg). Recent studies have demonstrated that in addition to the dysfunction of patient-derived Treg [1,2,3], T cells of MS patients are insensitive to the suppressive control through Treg [4,5]. This phenomenon termed Treg resistance is evoked by an accelerated IL-6 production, elevated IL-6 receptor expression and enhanced phosphorylation of protein kinase B (PKB)/c-Akt [4].

The influence of different approved therapies in MS such as interferon-beta (IFN-β) on restoring T cell functions, especially with regard to Treg suppression has not yet been addressed. Although the impact of IFN-β therapy on disease activity and its therapeutic efficiency is controversially discussed, IFN-β is the first-line treatment for patients with a relapsing-remitting MS [6,7,8]. A majority of autoimmune diseases are treated with biological drugs that specifically block cytokine receptors or signaling pathways including monoclonal antibodies against TNF-α (*i.e.*, Humira) [9,10] or the anti-IL-6 receptor (*i.e.*, Tocilizumab) [11,12] which are both employed for the treatment of rheumatoid arthritis. A critical disadvantage of these drugs is that they weaken the immune system and thereby increase the risk of opportunistic infections [13]. Thus, biologicals that specifically interfere with disease-associated transcription factors, receptors and molecules would be beneficial instead of using general immune suppression. Strategies rendering T cells responsive against regulation in combination with therapeutic strategies that enhance remyelination will improve future therapeutic concepts [14,15,16].

There is a large discrepancy between the desire for new specific therapeutics and the possibility to test these drugs *in vivo* [17,18,19]. Until today such analysis were restricted to *in vitro* studies or were addressed in mouse models.

Several potential drugs were identified in the experimental autoimmune encephalomyelitis (EAE), the mouse model for MS, but translational studies exhibited only minor success rates [20,21]. To overcome limitations of such experimental mouse models, “humanized mice” have been developed. The transfer of human cells into immunodeficient mice allows the modulation of human immune responses *in vivo* [22,23]. In these mice, Zayoud *et al.* detected antigen-specific responses of human T cells *in vivo* after the transfer of human PBMC from healthy donors (HD) and subsequent subcutaneous immunization with myelin oligodendrocyte glycoprotein [24].

Here, we studied the influence of IFN-β therapy on T cell immune regulation in regard to Treg control and observed that MS-related impaired suppression of T cells through Treg was significantly restored following IFN-β treatment both *in vitro* and *in vivo*. Furthermore, our results demonstrate that humanized mice are a beneficial tool to examine T cell-mediated immune responses of patients and their modulation by relevant therapeutics *in vivo*.

## 2. Results

### 2.1. T Cells in the Peripheral Blood of MS Patients Are Treg-Resistant

Studies on the activation and regulation of human T effector cells of autoimmune patients can exclusively be carried out *in vitro*. The significance of these results for complex interactions of human autoaggressive immune cells *in vivo* is limited. Especially, modern therapeutics for the targeted modulation are often epitope and species-specific. Therefore, we focused on the evaluation of a humanized mouse model allowing analysis and modulation of autoaggressive T cells from MS patients *in vivo*. We further investigated the influence of IFN-β treatment on MS-related Treg resistance.

**Figure 1:**
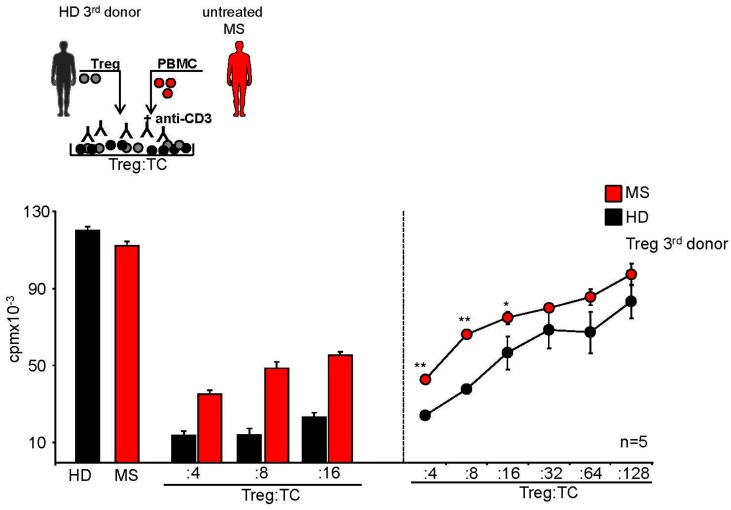
Treg resistant T cells of MS patients escaped control of functional active Treg. Treg-depleted PBMC from therapy-naive MS patients (MS, red) or healthy donors (HD, black) were cocultured with or without Treg and stimulated with anti-CD3 mAb. Proliferation was determined by 3H-Tdr incorporation on day three and displayed as mean ± SEM of triplicate measurements. (**Left**) bars represent mean ± SEM of triplicates of one representative experiment (of *n* = 28); (**Right**) curves show proliferation in presence of different Treg numbers of five different donors, *p*-values relative to T cell proliferation of HC (healthy control) * *p* < 0.05, ** *p* < 0.01 are shown.

Several groups have demonstrated that the suppressive capacity of CD25^+^Foxp3^+^ Treg from MS patients is impaired [1,3,25]. In addition, using classical *in vitro* suppressor assays, we observed that CD4^+^ and CD8^+^ T cells from therapy-naive MS patients were resistant to Treg-mediated suppression of independent third healthy individuals when compared to T cells from HD (Figure 1). Interestingly, this feature was independent of the diseases course, because no differences were observed between MS patients in relapse in comparison to those with a stable disease course (Supplemental Table S1).

**Figure 2:**
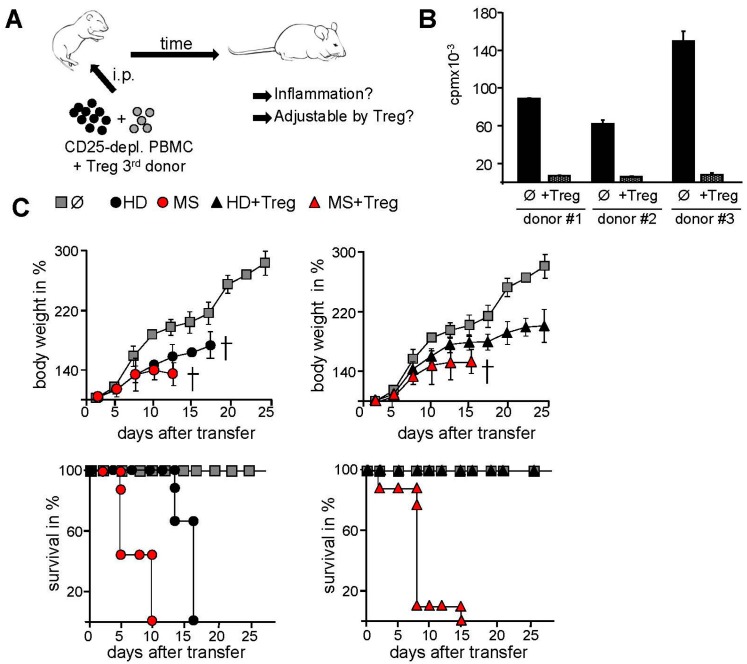
Transferring human PBMC of MS patients into newborn immunodeficient mice resulted in a severe systemic inflammation without protection by activated Treg. (**A**) Scheme: Treg were depleted within PBMC and replaced by the same amount of Treg from an independent HD. Subsequently 5 × 10^6^ CD25-depleted PBMC were transferred intraperitoneally into newborn immunodeficient mice; (**B**) To control Treg function we cocultured them with allogeneic PBMC (ratio 1:1) and stimulated with anti-CD3 mAb. T cell proliferation was determined by ^3^H-Tdr incorporation on day three and displayed as mean ± SEM of triplicate measurements. Three donors that were used in *in vivo* experiments are shown; (**C**) Treg were depleted within PBMC of HD (black) or MS (red) and replaced with the same amount of Treg from an independent 3rd HD. Subsequently, 5 × 10^6^ cells were injected with or without gp120 (5 μg/mouse) intraperitoneally into newborn NOD/*Scid*γc*^−^*^/*−*^ mice. Untreated mice served as controls (*n* = 5). Each point represents the cumulative mean weight of one group (five mice per group). Lower panel shows survival of mice in Kaplan-Meier plot till day 25. One of three independent experiments is shown. Cross means that all mice are dead.

### 2.2. Transfer of PBMC from Therapy-Naive MS Patients into Immunodeficient Mice Resulted in an Accelerated Systemic Inflammation that Is Not Controlled by Activated Treg

Thus, hyperactivated T cells in peripheral blood of therapy-naive MS patients are inefficiently controlled by functional active Treg. To further analyze this phenomenon *in vivo*, we transferred PBMC from MS patients or HD into immunodeficient mice. Intraperitoneal injection of human PBMC into newborn immunodeficient mice resulted in development of a lethal graft-versus host disease causing death within 15–30 days depending on the mouse strain and the number of transferred PBMC [26,27,28]. The T cell-dependent inflammatory response was characterized by decelerated growth, reduced body weight, reduced mobility and ruffled fur with a total mortality of over 95% [27,28]. This systemic inflammation could be controlled by polyclonal activation of intrinsic Treg via CD4-binding of the HIV protein gp120 [26,29,30]. Patient derived Treg were replaced by the same amount of Treg from an independent third healthy donor to exclude confounding effects of MS-related impaired functionality of Treg (Figure 2A). Treg function was validated in standard suppression assay (Figure 2B). Since suppression of activated Treg is antigen-nonspecific and donor-independent, functional active third donor Treg should also suppress the activation of human inflammatory T cells *in vivo* resulting in disease prevention. Indeed, systemic inflammation after HD PBMC transfer into immunodeficient NOD/*Scid*γc*^−^*^/*−*^ mice was prevented by activated third donor Treg, whereas mice engrafted with PBMC of therapy-naive MS patients developed an accelerated course of systemic inflammation. Mice showed early lethality that could not be ameliorated by activated Treg demonstrating that Treg resistance of MS T effector cells also occured *in vivo* (Figure 2C). Despite the injection of functional active Treg, all mice died within 18 days, demonstrating an increased aggressive activity of MS T effector cells and their resistance to Treg-mediated control.

### 2.3. MS-Related Treg Resistance Was Mediated by IL-6

**Figure 3:**
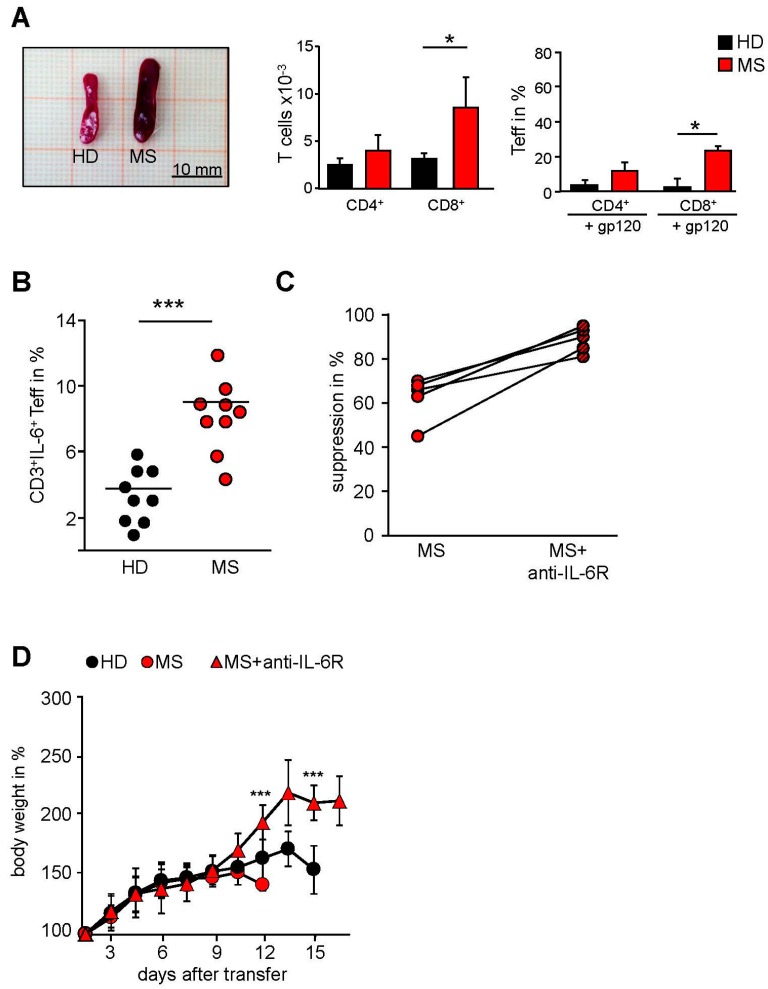
*In vivo* blockade of the IL-6 receptor by Tocilizumab ameliorated systemic inflammation. (**A**) Treg were depleted within PBMC of HD (black) or MS (red) and replaced with the same amount of Treg from an independent HD. Subsequently, 5 × 10^6^ cells were injected with or without gp120 (5 μg/mouse) intraperitoneally into newborn NOD/*Scid*γc*^−^*^/*−*^ mice. Seven days after transfer spleens were removed and proportion of CD4^+^ and CD8^+^ T cells was analyzed by flow cytometry; *p*-values relative to mice injected with HD-PBMC: * *p* < 0.05; (**B**) MS PBMC were cultured *in vitro* with Treg and stimulated with anti-CD3 mAb in presence (striped) or absence (red) of anti-IL6R mAb. Dots show percentage of suppression in presence of Treg normalized to PBMC alone (*n* = 5), *** *p* < 0.001; (**C**) Treg were depleted within PBMC of HD (black) or MS (red) and replaced with the same amount of Treg from an independent HD. Subsequently, 5 × 10^6^ cells were injected with or without gp120 (5 g/mouse) intraperitoneally into newborn NOD/*Scid*γc*^−^*^/*−*^ mice. Seven days after transfer spleens were removed and IL-6 production of reisolated T cells was determined by flow cytometry. Each point represents an individual mouse, line indicates mean value; (**D**) Treg were depleted within PBMC of HD (black) or MS (red) and replaced by the same amount of Treg from an independent HD. Subsequently, 5 × 10^6^ cells were injected with or without 10 g/mouse anti-IL-6R (Tocilizumab) intraperitoneally into newborn NOD/*Scid*γc*^−^*^/*−*^ mice. Each point represents the cumulative mean weight of one group (five mice per group). Two of three independent experiments are shown. *** *p* < 0.001.

We further investigated the distribution of human T cells in spleens of mice after disease onset. Systemic inflammation was associated with the accumulation of a large number of human CD4^+^ and CD8^+^ T cells in the spleen (Figure 3A). In line with accelerated inflammation following transfer of MS PBMC, we detected enlarged spleens (splenomegaly) (Figure 3A, left) with enhanced frequencies of human CD8^+^ T effector cells in comparison to mice administered with HD PBMC. Polyclonal activation of Treg significantly reduced the accumulation of splenic T effector cells after transfer of HD PBMC. In contrast, expansion of inflammatory T effector cells derived from MS patients could not be inhibited by activated Treg revealing an insensitivity of aggressive MS T effector cells towards Treg-mediated suppression *in vivo* (Figure 3A, right).

We recently observed that Treg resistance of freshly isolated MS T cells *ex vivo* was associated with an accelerated IL-6 production [4,5] and that blockade of IL-6 signaling restored MS T cells responsiveness (Figure 3B).

Re-isolated MS T cells exhibited significantly increased IL-6 production compared to T cells from diseased mice transferred with HD PBMC (Figure 3C). These results suggested that the accelerated course of systemic inflammation could be mediated by an enhanced IL-6 synthesis and that this disturbed IL-6 production in T cells of MS patients could be responsible for their *in vivo* Treg resistance. To confirm our hypothesis we consequently blocked IL-6 signaling *in vivo* using the clinically approved anti-IL-6R antibody Tocilizumab. A single dose of this antibody together with transferred MS PBMC significantly improved body weight and survival of mice, demonstrating that MS Treg resistance *in vivo* was largely influenced by IL-6 (Figure 3D).

**Figure 4:**
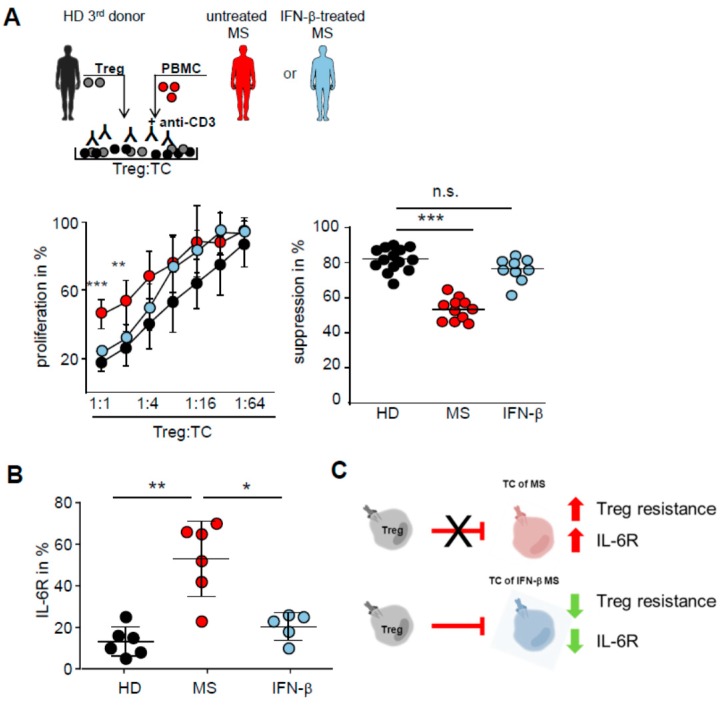
IFN-β therapy restores T cell responsiveness to Treg control. (**A**) Treg-depleted PBMC from therapy-naive (red), IFN-β-treated (blue) MS patients or HD (black) were cocultured with or without Treg from an independent HD and stimulated with anti-CD3 mAb. Proliferation was determined by 3H-Tdr incorporation on day three. Middle: curves show percentage of proliferation in presence of different Treg numbers normalized to proliferation of T cells alone as mean ± SEM (*n* = 11), *p*-values relative to T cells of HD, ** *p* < 0.01, *** *p* < 0.001 are shown. (Right) each point represents percentage of suppression of one independent donor (*n* = 11), *p*-values relative to suppression of HD, *** *p* < 0.001 are shown, n.s. means no significance; (**B**) IL-6R expression within PBMC from HD (black), therapy-naive (red), or IFN-β-treated (blue) MS-patients was determined by flow cytometry. Each point represents percentage of IL-6R^+^ cells within CD3^+^ cells of six independent donors, *p*-values relative to IL-6R expression of therapy-naive MS ** *p* < 0.01, * *p* < 0.05 are shown; (**C**) Shown is a graphic overview over the impact of IFN-β therapy on T cell function. IFN-β therapy reduces the IL-6R expression on T cells and further renders them sensitive against Treg-mediated suppression.

### 2.4. IFN-β Therapy Normalizes IL-6R Expression and Restores T Tell Responsiveness to Treg

Since we found that T cells of MS patients are Treg resistant we investigated the influence of IFN-β on T effector cell function. IFN-β is a widespread first line therapy used for relapsing remitting MS, but the underlying mechanisms remain unclear [6,31,32]. In particular its influence on T cell function with respect to Treg control is not resolved. Therefore, we cocultured Treg-depleted PBMC from therapy-naive or IFN-β-treated MS patients (PBMC from healthy volunteers served as control) with isolated Treg of an independent third donor and stimulated them with anti-CD3 mAb (Figure 4A, upper panel). Patients with various IFN-β medications were included in this study (Supplemental Table S1). As already shown in Figure 1, polyclonal activated Treg suppress T cell proliferation is donor independent and we observed that T cells from therapy-naive MS patients are insensitive to Treg control. Strikingly, proliferation of T cells isolated from MS patients treated with IFN-β, regardless of which preparation of IFN-β was used, were suppressed by activated Treg from independent third donors (Figure 4A, lower panel), suggesting that this immune-modulatory therapy at least partially restored Treg sensitivity of T cells in MS patients. In accordance with this finding expression of IL-6R on T cells from IFN-β-treated MS patients was significantly diminished compared to therapy-naive MS patients (Figure 4B), suggesting that IFN-β therapy targets IL-6 signaling and thereby renders T cells sensitive to Treg control (Figure 4C).

### 2.5. T Cells from IFN-β Treated MS Patients Showed an Improved Responsiveness for Immune Suppression in Vivo

**Figure 5:**
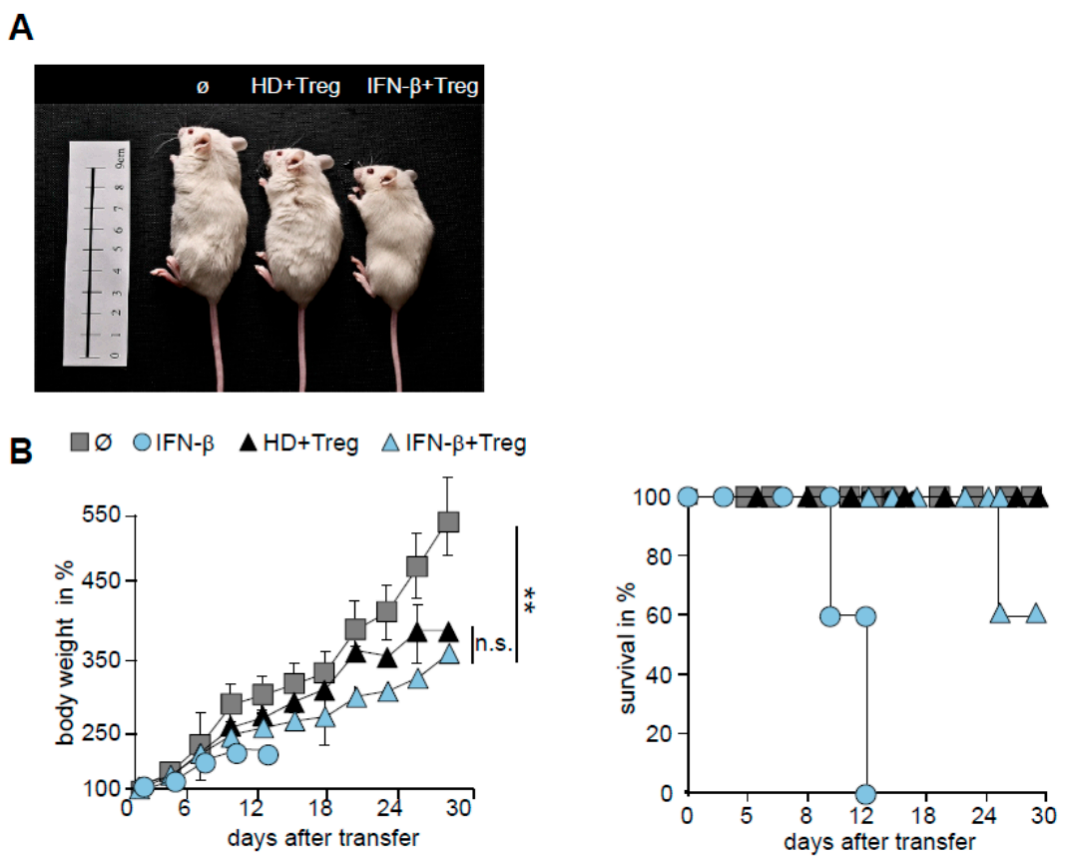
IFN-β therapy renders T cells responsiveness against suppressive function of Treg *in vivo*. (**A**) Shown are mice 20 days after transfer of PBMC from HD or IFN-β-treated MS patients with injection of gp120 compared with untreated mice; (**B**) Treg were depleted within PBMC of HD (black) or IFN-β-treated MS patients (blue) and replaced with the same amount of Treg from an independent HD. Subsequently, 5 × 10^6^ cells were injected with or without gp120 (5 g/mouse) intraperitoneally into newborn NOD/*Scid*γc^−/−^ mice. Untreated mice served as controls (*n* = 5). Each point represents the cumulative mean weight of one group (five mice per group), *p*-values relative to mean body weight of mice administered with PBMC from IFN-β-treated MS patients with Treg * *p* < 0.05, ** *p* < 0.01 are shown. n.s. means no significance. (Right) survival of mice in Kaplan–Meier plot within 30 days is shown. One of four independent experiments is shown.

These *in vitro* results suggested that the disturbed T cells function of therapy-naive MS patients was largely restored after IFN-β therapy. Furthermore, we investigated the susceptibility of IFN-β-treated MS T cells to Treg-mediated suppression *in vivo*. *In vitro* findings could also be reflected *in vivo*: transfer of PBMC from IFN-β-treated MS patients conducted in a milder systemic inflammation. Moreover, lethality of systemic inflammation was both significantly reduced and an increase in body weight was observed after Treg activation *in vivo* (Figure 5A,B). In summary, our data show that IFN-β therapy improves responsiveness of autoaggressive T cells to Treg-mediated suppression in MS patients.

## 3. Discussion

In the current study, we investigated the influence of IFN-β therapy on the responsiveness of T cells to Treg-mediated suppression in MS patients. We made the striking observation that T cells in therapy-naive MS patients are largely insensitive to Treg suppression whereas IFN-β treatment restored T effector cell susceptibility and IL-6 receptor expression significantly. In accordance to our *in vitro* observations, Treg resistance and impaired IL-6 production was also observed *in vivo* following transfer of immune cells from therapy-naive MS patients into immunodeficient mice. Moreover, severity of disease and lethality were largely improved after IFN-β therapy and T cells again responded to Treg control.

Dysregulation of T cell function occurs in a variety of autoimmune diseases but is attributed to diverse mechanisms [2,33,34,35]. Deregulated T cell responses in diabetes patients are believed to result from functionally altered antigen-presenting cells [33]. Among other explanations, inflammatory responses in MS have been attributed to impaired Treg function [36,37,38]. In addition, we and others found that Treg resistance in MS patients is mediated by T cells occurring independent from patient-derived Treg but with well documented alterations in antigen-presenting cells [4,5,39,40].

Since the 90s, IFN-β therapy is considered as a basic treatment in MS, although long-term studies demonstrated restricted effectiveness and the exact mechanisms are incompletely understood [32,41,42,43]. In suppression assays T cells from IFN-β-treated MS patients responded significantly better to Treg-mediated suppression than T effector cells of therapy-naive MS patients. Recently, several working groups linked the observed Treg resistance in therapy-naive MS patients with an altered IL-6 production [4,5,44]. We further observed that the IFN-β treatment decreased IL-6 receptor expression on T cells thereby inhibiting the IL-6 signaling cascade and rendering T cells again responsive to Treg. This is in line with *in vitro* studies demonstrating that IFN-β inhibited T cell proliferation and impaired up-regulation of IL-2 receptor alpha-chain, CD2 and transferrin receptor on activated T effector cells [45,46]. The limited therapeutic effect of IFN-β in MS may be attributed by our observation that IFN-β restores mainly T cell sensitivity to Treg function. More over MS is a multifactorial autoimmune disease that affects several immune cells and tissues. We therefore propose that a combined treatment approach addressing T cell responsiveness and Treg dysfunction might be beneficial.

A variety of autoimmune diseases are associated with an excessive IL-6 production [47,48,49]. Therefore, blocking agents directed towards IL-6 or the IL-6 receptor might be a possible strategy to treat autoimmune diseases. Interestingly, Tocilizumab an anti-IL-6 receptor antibody is already used as an effective drug for the treatment of patients with rheumatoid arthritis and juvenile idiopathic arthritis [11,50].

Furthermore, cytokine-directed treatments such as IFN-β or Tocilizumab are associated with serious adverse events [7,51,52]. Since these therapies also suppress essential adaptive immune mechanisms treated patients are also prone to opportunistic infections [13]. There is a need for more selective treatment strategies, which require specific test systems to analyze their efficiency and their mode of action. Application of these approaches is largely based on studies in rodent models [20,53]. The experimental autoimmune encephalomyelitis has been used for decades in research as a mouse model to study MS and gave rise to a variety of important mechanisms in the pathogenesis of MS [20,21,54]. However, rodent disease models are also limited [17] indicated by the fact that therapies yielding promising results in the mouse model achieved only disappointing success in clinical trials [20,21,55]. For example, depleting T cell antibodies identified in the EAE model showed no significant beneficial effects in the clinic [56]. Furthermore, data generated in mice cannot be directly adjusted into humans due to numerous species-specific differences in the immune system between mouse and men and epitope-specific biologicals (*i.e.*, anti-human cytokine antibodies) cannot be functionally tested in mice [17].

Improved animal models, which are related to the human immune system, are therefore always required and developed [18,23,57]. These so called humanized mouse models might build a bridge between *in vitro* studies and common mouse models. The course of humanization is strongly dependent on the type and number of transferred cells, mouse strains and age of animals that were used [26,27,28,58].

Adoptive transfer of human PBMC into newborn immunodeficient mice as used in the current study leads to the induction of systemic inflammation induced by human immune cells. The disease is a T cell-mediated immune response that is triggered by human T cells *in vivo*. The polyclonal CD4-mediated activation of Treg prevents disease induction [28,29,30]. Therefore the model is suitable to study the regulation and modulation of human T cells *in vivo*. More important, this model allows the investigation of transferred patient-derived immune cells.

Transferring peripheral immune cells from therapy-naive MS patients into newborn immunodeficient mice led to an accelerated infiltration of human T cells into various organs and resulted in a lethal systemic inflammation. Due to the observed *in vitro* Treg resistance of MS T cells [4,5], it was not surprising that CD4-mediated Treg activation could not prevent the induction of systemic inflammation *in vivo*. Treg resistance of MS T cells in humanized mice is IL-6-dependent, as the single administration of the IL-6R blocking mAb Tocilizumab restored Treg-mediated suppression, mitigated course of systemic inflammation and ensured survival of the mice.

Restoration of T cell function after IFN-β treatment was also reflected *in vivo*: immunodeficient mice that received immune cells from IFN-β-treated patients together with activated Treg from independent HD showed a milder disease course and decreased lethality.

The use of adult mice or transfer of smaller PBMC numbers delayed GvHD induction or prevented it completely over a period of time [27,58]. Martin *et al.* for example used adult immunodeficient mice and transferred PBMC from allergic donors to birch pollen and subsequently induced by allergen airway challenge an airway inflammation and airway hyper responsiveness [27]. Treatment with gp120 prior to allergen challenge abrogated airway hyper responsiveness and reduced the inflammatory immune response.

Our results demonstrated that adoptive transfer of human immune cells into immunodeficient mice allows their functional analysis and modulation by different biologicals *in vivo*. Although the causes that lead to the development of MS cannot be determined in this transfer model, it provides insights into the efficacy of MS treatment and mode of action analyses of individual therapeutics.

An additional model for *in vivo* investigation of human immune responses is the “fully humanized mouse”. By application of human hematopoietic stem cells into newborn immunodeficient mice a nearly complete human immune system is formed [59,60]. The induction of a MS-like disease in these mice might give the opportunity to study disease induction, MS pathology and treatment opportunities *in vivo*.

## 4. Material and Methods

### 4.1. Patients and Healthy Controls

Eighty-five patients with a relapsing-remitting course (RRMS, age 18 to 64 years) and 4 patients with a clinically isolated syndrome (CIS, age 22 to 53) fulfilling the revised McDonald criteria for multiple sclerosis [49] and were included in this study. Thirty-one patients were treated for at least 4 months with IFN-β. Fifty-eight patients had not received previous treatment or immunosuppressive agents six months before time point of analysis and were clinically stable. PBMC from healthy controls served as controls. PBMC were isolated within 12 h after blood collection and were used directly in *in vitro* or *in vivo* experiments. Blood was kept at room temperature before PBMC enrichment. According to the principles expressed in the Helsinki Declaration and to the ethics committee-approved protocols patients provided written informed consent before participating in this study. This study was approved by the local Ethics Committee (Landesärztekammer Rhineland Palatine No. 837.019.10 (7028), approved on 4 March 2010.).

### 4.2. Transfer of Human Immune Cells

NOD/*Scid*γc^−/−^ mice were obtained at one to four days after birth from the central animal facility, Johannes Gutenberg University, Mainz. Experiments were performed in accordance with relevant laws and institutional guidelines. Systemic inflammation was induced as described before [26]. Briefly, 5 × 10^6^ peripheral blood mononuclear cells (PBMC) were injected intraperitoneally into newborn mice with/without 5 µg/mouse gp120 or 10 µg/mouse anti-IL-6R mAb (Tocilizumab). Untreated mice served as controls. Weight changes were monitored every second day. Results are presented as percent mean body weight ± SEM based on initial weight.

### 4.3. Culture Medium and Antibodies

Blood-derived human immune cells and human cells isolated from humanized mice were cultured in X-VIVO-15 (Lonza, Verviers, Belgium). Flow cytometric analysis was performed using the following antibodies. Anti-human CD3 (SK7), anti-human CD3 (UCHT1), anti-human CD4 (RPA-T4), anti-human CD8 (SK1), anti-human CD14 (M5E2), anti-human CD19 (HIB19), anti-human CD25 (M-A251), anti-human IL-6 (MQ2-13A5), anti-(pS473) pPKB/c-Akt (M89-61), all from BD Pharmingen (Heidelberg, Germany) anti-human CD8 (BW 135/80, Miltenyi Biotec, Bergisch Gladbach, Germany), Fluorokine^®^ biotinylated human Interleukin-6 (R&D systems, Minneapolis, MN, USA). Cell viability during flow cytometric analysis was determined using 7-AAD (eBioscience, San Diego, CA, USA).

### 4.4. Flow Cytometry

For surface staining of PBMC or isolated T cells indicated antibodies were incubated for 30 min at 4 °C and washed twice with PBS. Stained cells were measured on LSRII with FACS Diva Software (BD Bioscience, Heidelberg, Germany). To detect phosphorylated PKB/c-Akt, cells were fixed at 37 °C (BD Cytofix™ Buffer); permeabilized (BD™ Phosflow Perm Buffer, Heidelberg, Germany), washed twice with BD Pharmingen Stain buffer and stained for the indicated antibodies according to the manufacturer’s instructions.

### 4.5. Isolation of T Cell Subsets

CD4^+^CD25^+^Foxp3^+^ Treg were isolated from PBMC using anti-CD25 MicroBeads (Miltenyi Biotec) and depleted of contaminating CD8^+^, CD14^+^ and CD19^+^ cells with Dynabeads (Invitrogen, Hamburg, Germany) as described previously [61]. Purity was routinely >80%, Treg functionality was ensured in standard suppressor assay. For some experiments PBMC were depleted of CD3 or CD25 using corresponding Dynabeads (1 bead/cell; Invitrogen, Hamburg, Germany).

### 4.6. Cytokine Analysis

For intracellular cytokine staining anti-IL-6-APC was used. Spleen cells from humanized mice were activated with 1 g/mL Ionomycin and 1 ng/mL PMA for 5, 4 h in the presence of Monensin (1.3 M/mL). After stimulation cells were collected, washed, permeabilized (perm/fix solution; BD Pharmingen, Heidelberg, Germany) and stained for above-mentioned cytokine.

### 4.7. Suppressor Assays

Treg-depleted PBMC (10^5^ cells) were stimulated with 0.5 g/mL anti-CD3 mAb (OKT3) and cultured in presence or absence of different Treg ratios (Treg: TC 1:1 to 1:64) [30,61]. T cell proliferation was determined on day three of cultures by addition of 37 kBq/well ^3^H-Tdr for additional 16 h.

### 4.8. Cell Isolation from Different Tissues of Humanized Mice and MLR

Spleen, lymph node and peripheral blood were harvested at indicated time points and analyzed for infiltration of human immune cells. Organs were homogenized through a cell strainer (100 m; BD Bioscience). Erythrocytes were lysed and single cell preparations were used for flow cytometry or adopted into suppression assays. Cells were counted and stained for human CD45^+^ immune cells. 1 × 10^5^ CD45^+^ cells were stimulated with anti-CD3 mAb and cultured in presence or absence of different human Treg ratios. Proliferation was determined on day three.

### 4.9. Statistical Analysis

Results represent means ± SEM. Statistical significance was determined using unpaired Student’s *t* test relative to HD. *p*-values of less than 0.05 were considered significant and indicated in the corresponding figures (* *p* < 0.05; ** *p* < 0.01; *** *p* < 0.001). For some experiments statistical significance was determined by Mann–Whitney Test. *p*-values of less than 0.05 were considered significant and indicated in the corresponding figures (* *p* < 0.05; ** *p* < 0.01; *** *p* < 0.001).

## 5. Conclusions

In summary, our results provide strong evidence that IFN-β therapy ameliorated T effector cell function in regard to Treg-mediated suppression due to diminished IL-6R expression on T cells in an *in vivo* mouse model. The results show that humanized mice can be used to build a bridge between *in vitro* assays and established mouse models.

## Supplementary Materials

Supplementary materials can be found at http://www.mdpi.com/1422-0067/16/07/16330/s1.
