# Supplementary material for: Interferon-Beta Therapy of Multiple Sclerosis Patients Improves the Responsiveness of T Cells for Immune Suppression by Regulatory T Cells
Source: Int J Mol Sci. 2015 Jul 17;16(7):16330–46. doi: 10.3390/ijms160716330 (PMC4519953; doi:10.3390/ijms160716330)
Supplement: Supplementary file 1 [file ijms-16-16330-s001.pdf]

Supplementary Information

Table S1. Clinical characteristics of multiple sclerosis patients.PBMC were collected in heparinized tubes from 85 patients with a relapsing-remitting course (RRMS. age 18 to 64 years). 17 patients with RRMS showed a relapse. remaining patients were in remission. Expanded Disability Status Scale (EDSS) was used to quantify disability (0–6). We did not detect any differences in T cell responses regarding the course of disease. 30 patients were treated for at least 4 month with IFN-β therapy (Rebif 22. 44. Betaferon. Plegridy. Avonex). Other patients had not received previous treatment or immunosuppressive agents six months before time point of analysis and were clinically stable. According to the principles expressed in the Helsinki Declaration and to the ethics committee-approved protocols patients provided written informed consent before participating in this study.

Sex	Age (year)	Disease Course	Disease Duration (year)	Treatment	Level of Disability (EDSS)	State
M	53	RRMS	11.06	Rebif 44	6.00	Remission
F	31	RRMS	0.24	Rebif 22	2.00	Remission
M	35	RRMS	14.09	untreated	1.00	Remission
F	38	RRMS	2.09	untreated	2.00	Remission
F	25	RRMS	1.01	Rebif 44	1.00	Remission
F	30	RRMS	3.11	untreated	3.00	Relapse
M	37	RRMS	2.40	Rebif 44	3.00	Remission
F	52	RRMS	0.57	untreated	1.00	Remission
F	52	RRMS	7.16	Rebif 22	4.00	Remission
F	27	RRMS	0.46	untreated	4.00	Remission
M	25	RRMS	3.24	untreated	2.00	Remission
M	55	RRMS	33.25	untreated	6.00	Remission
M	31	RRMS	0.35	untreated	1.00	Remission
M	31	RRMS	4.27	Rebif 44	3.00	Relapse
M	46	RRMS	0.60	untreated	3.00	Remission
F	36	RRMS	1.69	untreated	2.00	Remission
F	35	RRMS	2.37	untreated	1.00	Remission
F	40	RRMS	0.63	untreated	2.00	Remission
F	64	RRMS	20.39	untreated	4.00	Remission
F	45	RRMS	0.94	untreated	3.00	Remission
F	52	RRMS	0.94	untreated	1.00	Remission
M	36	RRMS	14.53	untreated	1.00	Remission
F	40	RRMS	0.68	untreated	4.00	Remission
M	27	RRMS	0.85	untreated	1.00	Relapse
M	26	RRMS	1.62	untreated	2.00	Relapse
F	33	RRMS	7.77	untreated	3.00	Remission
F	41	RRMS	18.78	untreated	5.00	Remission
F	52	RRMS	1.29	untreated	1.00	Remission
F	37	RRMS	9.96	untreated	3.00	Remission

Table S1. *Cont.*

Sex	Age (year)	Disease Course	Disease Duration (year)	Treatment	Level of Disability (EDSS)	State
M	32	RRMS	0.22	untreated	2.00	Remission
F	47	RRMS	21.98	untreated	6.00	Remission
F	53	RRMS	1.49	untreated	1.00	Remission
F	28	RRMS	1.33	Rebif 44	3.00	Remission
F	24	RRMS	7.13	untreated	1.00	Remission
F	29	RRMS	4.13	untreated	4.00	Remission
F	25	RRMS	1.63	untreated	2.00	Remission
F	34	RRMS	8.65	untreated	5.00	Remission
F	27	RRMS	0.25	untreated	2.00	Remission
M	40	RRMS	11.17	untreated	3.00	Remission
M	21	RRMS	1.46	untreated	2.00	Remission
F	26	RRMS	2.13	Rebif 44	1.00	Remission
M	39	RRMS	12.23	untreated	6.00	Remission
M	48	RRMS	14.26	untreated	2.00	Relapse
F	34	RRMS	8.33	untreated	3.00	Remission
F	18	RRMS	0.69	untreated	1.00	Remission
F	53	RRMS	2.14	untreated	1.00	Remission
F	32	RRMS	3.97	untreated	1.00	Remission
M	32	RRMS	1.88	untreated	1.00	Remission
F	44	RRMS	20.35	Rebif 44	3.00	Remission
F	29	RRMS	0.41	Rebif 22	1.50	Remission
F	41	RRMS	4.41	untreated	3.00	Relapse
M	49	RRMS	0.13	untreated	3.00	Relapse
F	22	RRMS	5.10	Rebif 44	3.50	Remission
F	29	RRMS	3.49	Rebif 22	3.00	Remission
F	25	RRMS	0.60	untreated	1.00	Remission
F	25	RRMS	4.69	Rebif 44	2.00	Remission
F	30	RRMS	17.69	untreated	3.00	Relapse
M	32	RRMS	0.20	untreated	3.00	Relapse
F	55	RRMS	2.79	untreated	2.00	Relapse
F	19	RRMS	1.62	Rebif 44	1.00	Remission
F	32	RRMS	13.83	untreated	4.00	Remission
F	55	RRMS	2.84	untreated	2.00	Relapse
M	30	RRMS	3.84	untreated	1.00	Relapse
F	30	RRMS	3.88	untreated	1.00	Remission
M	22	CIS	0.12	untreated	2.00	Relapse
F	49	RRMS	3.15	untreated	2.00	Remission
F	33	RRMS	16.16	Betaferon	1.00	Remission
F	23	RRMS	0.07	untreated	3.00	Relapse
F	33	RRMS	5.24	Rebif 44	1.00	Remission
M	35	RRMS	1.49	Rebif 44	2.00	Remission
M	28	RRMS	1.59	Rebif 44	0.00	Remission

Table S1. *Cont.*

Sex	Age (year)	Disease Course	Disease Duration (year)	Treatment	Level of Disability (EDSS)	State
F	23	RRMS	6.34	Rebif 44	1.00	Remission
F	45	RRMS	8.39	Rebif 44	2.00	Remission
F	53	RRMS	15.47	untreated	3.00	Relapse
M	46	RRMS	5.54	untreated	4.00	Remission
M	28	RRMS	0.54	untreated	1.00	Relapse
M	33	RRMS	13.85	untreated	1.00	Remission
F	26	RRMS	4.72	Plegridy	1.00	Remission
F	45	RRMS	0.14	untreated	1.00	Relapse
F	24	RRMS	0.59	Rebif 44	3.00	Remission
M	23	RRMS	1.32	Rebif 44	1.00	Remission
M	44	RRMS	2.27	Rebif 44	2.00	Remission
F	38	RRMS	16	Rebif 44	1.00	Remission
F	26	RRMS	5	Betaferon	1.00	Remission
F	37	CIS	4	Avonex	1.00	Remission
F	35	CIS	4	Rebif 22	2.50	Remission
M	24	RRMS	4	Rebif 44	1.50	Remission
F	36	RRMS	5	Avonex	1.00	Remission
